# Role of Immune Checkpoint Inhibitors in Cervical Cancer: From Preclinical to Clinical Data

**DOI:** 10.3390/cancers13092089

**Published:** 2021-04-26

**Authors:** Simona Duranti, Antonella Pietragalla, Gennaro Daniele, Camilla Nero, Francesca Ciccarone, Giovanni Scambia, Domenica Lorusso

**Affiliations:** 1 Scientific Directorate, Fondazione Policlinico Universitario A. Gemelli IRCCS, 00168 Rome, Italy; simona.duranti@policlinicogemelli.it (S.D.); antonella.pietragalla@policlinicogemelli.it (A.P.); gennaro.daniele@policlinicogemelli.it (G.D.); camilla.nero@policlinicogemelli.it (C.N.); giovanni.scambia@policlinicogemelli.it (G.S.); 2 Department Woman and Child Health and Public Health, Division of Gynaecologic Oncology, Fondazione Policlinico Universitario A. Gemelli IRCCS, 00168 Rome, Italy; francesca.ciccarone@policlinicogemelli.it; 3 Department of Life Science and Public Health, Università Cattolica del Sacro Cuore, 00168 Rome, Italy

**Keywords:** cervical cancer, immunotherapy, immune checkpoint inhibitors, PD-1, PD-L1, CTLA4

## Abstract

**Simple Summary:**

Cervical cancer represents one of the main leading causes of cancer-related mortality in women worldwide. In contrast to patients with early-stage disease, those with advanced, recurrent, or metastatic cervical cancer have a poor prognosis and new treatment strategies are needed. Immunotherapy has recently modified the natural course of different tumors, such as melanoma and lung cancer. The aim of this review is to evaluate the possible role of immune checkpoint inhibitors in cervical cancer treatment.

**Abstract:**

Human papillomavirus (HPV) infection is the recognized cause of almost all cervical cancers. Despite the reduction in incidence due to a wide use of screening programs and a specific vaccine, the prognosis of cervical cancer remains poor, especially for late-stage and relapsed disease. Considering the elevated rates of PD-L1 expression in up to 80% of cervical cancers, a strong rationale supports the use of immunotherapy to restore the immune response against tumor. The aim of this review is to analyze the possible role of immune checkpoint inhibitors in cervical cancer treatment, with a particular focus on the rationale and on the results of phase I and II clinical trials. An overview of ongoing phase III studies with possible future areas of development is also provided.

## 1. Cervical Cancer Landscape

Cervical cancer (CC) is the fourth most frequent cancer in women and represents one of the leading causes of cancer-related mortality worldwide. It causes 342,000 deaths annually and mortality varies across different countries, from less than two per 100,000 in Australia/New Zealand to more than 22 per 100,000 in some African countries [1]. 

Human papillomavirus (HPV) infection is the recognized cause of the majority of CCs [2]. Despite the reduction of incidence with the use of screening programs and the introduction in clinical practice of a specific vaccine, the prognosis of this tumor remains poor, particularly for late-stage and relapsed disease. 

Patients with localized CC have a good prognosis, with a 5-year survival rate of 91%, while for patients with advanced or recurrent disease, the prognosis is poor, with a 5-year survival rate of 17% [3]. 

Stages IA1 can be managed with conisation or hysterectomy based on the fertility desire of the patient. In patients with FIGO stage IA2, IB, and IIA, the standard treatment is represented by radical hysterectomy with lymphadenectomy [4]. For patients with locally advanced CC, cisplatin-based chemoradiotherapy (CRT) has represented the standard of care for almost 2 decades [5,6,7,8,9,10]. However, the risk of disease recurrence is 10–20% for early stages and 50–70% for patients with locally advanced disease. Advanced and recurrent CC, not amenable of surgery or radiotherapy, has a poor prognosis with median progression free survival (PFS) of 2–5 months and overall survival (OS) of 5–16 months [11]. Cisplatin represents the standard of care, showing a response rate of 13% and 36% with monotherapy or platinum-based doublet, respectively [4,12,13,14,15]. Recently, the association of bevacizumab with chemotherapy in patients with metastatic CC showed an advantage in OS of 4 months compared with chemotherapy alone (17 months versus 13 months) and a response rate of 48% versus 36% [16]. However, in the case of disease progression, only limited treatment options are available and no standard of care chemotherapy regimen is defined for second-line [4,12,17]. The commonly used treatments are taxanes, topotecan, gemcitabine, and targeted therapy in the context of clinical trials, with a disappointing response rate of 13.2%, median PFS of 3.2 months, and median OS of 9.3 months [18].

Therefore, new treatment modalities and paradigms are needed to improve the prognosis of women diagnosed with CC. In this scenario, immunotherapy and immune checkpoint inhibitors (ICIs) in particular, may play a role in the treatment of cervical neoplasms in various settings of disease.

## 2. Immune Checkpoint Inhibitors and Cancer

In recent years, a revolution in the treatment of cancer has begun, owing to the identification of tumor-specific immune responses against tumors. Indeed, immunotherapy aims to stimulate the immune system with two different strategies: active and passive immunotherapy. The first mechanism involves the administration of cancer vaccines that stimulate the host’s immune system against malignant cells, while the second contemplates the use of exogenous immune compounds, such as ICIs or adoptive T cell therapy (ACT), that enhance the immune response against tumors [19].

Cytotoxic T lymphocyte antigen 4 (CTLA4) and programmed cell death 1 (PD-1) are the most common molecules targeted by ICIs. CTLA4 is a negative regulator of T cell activation and its inhibition allows T cells’ response against cancer [20]; likewise, also the PD-1 axis is involved in the negative T cell regulation and its inhibition leads to the recovery of the cytotoxicity of T cells towards tumors [21]. Ipilimumab, a human IgG1κ anti-CTLA4 monoclonal Antibody (mAb), which binds CTLA4 and blocks its interaction with ligand CD80/CD86, was the parent ICI approved in 2011 by the Food and Drug Administration (FDA) for first and second-line therapy of melanoma [22]. Since then, the European Medicines Agency (EMA) approved several PD-1/PD-L1 and CTLA4 inhibitors for the treatment of different solid tumors [23,24,25,26,27,28,29].

The potential impact of immunotherapy on cancer survival has been recently addressed by Emens et al. in a model showing that single-agent ipilimumab or PD-1/PD-L1 inhibitors and immunotherapy combinations targeting both CTLA-4 and PD-1 pathways are associated with long-term survival rates ranging from 10–30% and 50–60%, respectively [30]. 

The typical toxicity related to ICIs is the autoimmune toxicity, caused by an exaggerated immune effector response, which exceeds the limits of immune tolerance to self-tissues [31]. The incidence of immune-related adverse events is between 15–90% and those requiring treatment are reported in 15% and 30% of patients who receive PD-1/PD-L1 and CTLA4 inhibitors, respectively. Skin toxicity is the most frequent adverse event described in patiens treated both with CTLA-4 and PD-1/PD-L1 inhibitors. CTLA4 inhibitors are typically associated with gastrointestinal and cerebral autoimmune toxicities, whereas PD-1 pathway inhibitors may be associated with hypothyroidism, hepatoxicity, and pneumonitis. However, toxicities associated with immunotherapy are manageable in the majority of cases; nevertheless, in up to 55% of patients, grade 3–4 severe adverse events are reported, particularly when ICIs combinations are used [32].

## 3. Immune Checkpoint Inhibitors and Cervical Cancer

### 3.1. Rationale to the Use of Checkpoint Immunotherapy

Almost all cases of CC are caused by HPV infection. The HPV genome encode for seven early proteins (E1, E2, E4, E5, E6, E7, and E8) and two structural proteins (L1 and L2). The integration of viral DNA with the host’s genome causes the cancer development. The loss of the viral E2 gene leads to the upregulation of oncoproteins E6 and E7, which complexe with p53 and the retinoblastoma protein (pRb), thus causing an alteration of the cell cycle, genomic instability, and neoplasia [33,34]. At the first phase, HPV-positive cells inhibit acute inflammation and immune recognition, leading to an escape from immune system control and viral persistence. During the next phase, neoplastic cells promote chronic inflammation and interact with tumor microenvironment favoring carcinogenesis [35]. The relationship between HPV infection and inflammation is well known and a recent study in head and neck squamous cell carcinoma demonstrates that it could be responsible for the induction of PD-L1 expression [36]. The presence of PD-L1 and a T cell activation gene-expression signature, both representative of an inflammatory state of the tumor, are considered biomarkers predictive of response to immunotherapy. Indeed, high levels of PD-L1 expression were reported in 35–96% of CC [37,38,39], suggesting that PD-1 pathway may be an attractive therapeutic target for these patients, although its role as predictive biomarker is still conflicting [40,41,42,43].

Furthermore, as a consequence of viral origin, CC is associated with a specific immunologic profile and in about 20% of cases with high tumor mutational burden (TMB), an indicator of tumor antigenicity, thus potentially enabling a successful use of immunotherapy in this tumor [44], as reported for other TMB-high cancers. Moreover, Lazo et al. reported that 8% of CCs present microsatellite instability (MSI) [45], suggesting the possibility that this subgroup of patients may respond to treatment with ICIs [46].

All of these CC features (tumor inflammatory state, expression of PD-L1, high TMB and MSI) support the rationale for using immunotherapy in the treatment of this tumor. However, limited data are available about the use of ICIs in CC patients and a lot of studies evaluating these agents, both in monotherapy or in combination strategies, are currently ongoing.

### 3.2. Clinical Development

#### 3.2.1. Single-Agent Trials with PD-1/PD-L1 Inhibitors

To our knowledge, four early phase clinical trials were published testing the two different ICIs, pembrolizumab and nivolumab (Table 1).

Pembrolizumab was tested in two multicohort studies, the phase Ib trial Keynote-028 [40,41] and the phase II trial Keynote-158 [42]. The first study enrolled only PD-L1-positive patients (in total 24 CC patients), while in the second trial, 83.7% (82) of CC patients were PD-L1-positive. The schedule of the anti-PD-1 was different; in the Keynote-028 trial, patients received pembrolizumab 10 mg/kg every 2 weeks for up to 24 months, while in the Keynote-158 trial, enrolled patients received pembrolizumab 200 mg flat dose every 3 weeks for up to 2 years. In both cases, the primary endpoint was the overall response rate (ORR) and reached 17% and 12.2%, respectively. In the phase II trial, three complete responses (CR) and nine partial responses (PR) were reported, all in PD-L1-positive cancers. Median PFS and median OS were comparable in both studies, in particular median PFS was about 2 months and median OS was about 11 months, considering only PD-L1-positive patients in the phase II trial. Adverse events (AEs) were observed in 18 (75%) and 64 (65.3%) patients in phase I and II trials, respectively, but grade ≥3 treatment-related AEs were reported in 21% (five) of the Keynote-028 patients, where only grade 3 AEs were observed (neutropenia, rash, colitis, Guillain–Barrè syndrome, and proteinuria) and in 12.2% of the Keynote-158 patients, where the most common were increased alanine aminotransferase and aspartate aminotransferase. In both trials, no deaths were reported [40,41,42]. Based on the phase II trial, on 12 June 2018, the FDA approved pembrolizumab for the treatment of PD-L1-positive (CPS ≥ 1) recurrent or metastatic CC with disease progressing on or after chemotherapy [47].

Nivolumab was tested in two trials, the phase I/II CheckMate 358 [43] and the phase II NCT02257528/NRG-GY002 [48], with mixed results. The first study enrolled patients with HPV-positive cervical, vaginal, and vulvar cancers and, in total, 19 CC patients received nivolumab monotherapy 240 mg every 2 weeks for ≤2 years. The trial showed a promising ORR of 26.3%; three patients obtained a CR and two patients a PR. Median PFS was 5.1 months and median OS was 21.9 months. The majority of treatment-related AEs were grade 1 to 2 and the most common were gastrointestinal (21.1%) and skin (21.1%) reactions. Three grade 3 or 4 treatment-related AEs were reported (one gastrointestinal, one pneumonitis, and one hepatocellular injury). Of note, the responses were only reported in the CC cohort [43].

In the second trial, patients with persistent/recurrent CC received a different schedule of the anti-PD-1, in particular, nivolumab was administered at 3 mg/kg IV every 2 weeks until disease progression or intolerable toxicity. Twenty-six patients were enrolled in the trial and about 77% were PD-L1-positive. Objective response was the primary endpoint and reached only 4%, with one patient obtaining a confirmed PR and nine patients having SD (36%). Estimated PFS and OS at 6 months were 16% and 78.4%, respectively. Twenty-one (84%) patients reported a treatment-related AE and also in this study the majority were grade 1–2. Six (24%) patients had grade 3 treatment-related AEs, one of which discontinued treatment due to liver toxicity; two grade 4 and zero grade 5 treatment-related AEs occurred [48].

#### 3.2.2. Single Agent Trials with CTLA4 Inhibitor

The anti-CTLA-4 ipilimumab was tested in monotherapy in two early phase trials. The first trial was a phase I/II trial, where 42 patients with metastatic or recurrent CC received ipilimumab 3 mg/kg every 3 weeks for four cycles in the run in safety cohort and 10 mg/kg every 3 weeks for four cycles, followed by four cycles of maintenance therapy every 12 weeks in the phase II cohort. Only one PR was reported among the 34 evaluable patients. Median PFS was 2.5 months and median OS was 8.5 months. AEs were manageable and grade 3 treatment-related AEs included diarrhea and colitis [49]. 

Ipilimumab was further investigated in the phase I GOG 9929 trial, where it was administered as maintenance after chemoradiation in 34 CC patients with positive nodes. The anti-CTLA-4 was administered at three dose levels: 3 mg/kg, 10 mg/kg, and an expansion cohort of 10 mg/kg. The primary endpoints were the maximum tolerated dose (MTD), defined at 10 mg/kg, and dose-limiting toxicities (DLT). There were no significant late toxicities and 1-year disease-free survival (DFS) was 74% [50] (Table 1).

#### 3.2.3. Immune Checkpoint Inhibitors’ Combinations

The combination of treatments has traditionally been a mainstay of oncology and this concerns chemotherapy, target therapy, and immunotherapy. Recent studies showed that tumor-specific CD8+ T cells express several inhibitory receptors (including CTLA-4, PD-1, TIM-3, BTLA, and LAG3) and their double simultaneous blockade acts synergistically to render T cells more functional than a single blockade [51].

At the European Society of Medical Oncology (ESMO) 2019 meeting, the results of a trial exploring the combination of nivolumab plus ipilimumab at two different schedule were presented. Advanced/recurrent CC patients were randomized to nivolumab 3 mg/kg every two weeks and ipilimumab 1 mg/kg every 6 weeks (Combo A), or nivolumab 1 mg/kg and ipilimumab 3 mg/kg every 3 weeks for four doses followed by nivolumab 240 mg every 2 weeks (Combo B), for ≤24 months. The results were encouraging, mostly in patients who had not received previous treatment; in particular, ORR was 46% in Combo B versus 32% in Combo A for patients without previous treatment and 36% versus 23%, respectively, in pretreated patients. In Combo A, median PFS was 13.8 and 3.6 months in patients without and with previous treatment, respectively, whereas in Combo B, median PFS was 8.5 months and 5.8 months, respectively. OS was NR in both combinations for non-pretreated patients and 10.3 months and 25.4 months in previously treated patients for Combo A and Combo B, respectively. Incidence of grade 3–4 treatment-related AEs was 28.9% in Combo A and 37.0% in Combo B, respectively. No new safety signals were reported [52] (Table 1).

Another checkpoint inhibitor, the anti-PD-L1 durvalumab, is under evaluation in a phase I trial in combination with the anti-CTLA-4 tremelimumab. Thirteen CC patients who failed standard treatment were enrolled: in this trial, no response was reported and six (46.2%) patients experienced SD. The majority of treatment-related AEs were grades 1 and 2. AEs ≥ grade 3 were reported in 12 patients, mainly represented by diarrhea and colitis (n = 5). There was one grade 5 treatment-related AE (multi organ failure) [53] (Table 1).

Finally, at ESMO 2020, the results of two independent, parallel phase II trials evaluating the anti-PD-1 balstilimab alone or in association with the anti-CTLA-4 zalifrelimab in recurrent and advanced CC were reported. One hundred sixty-one and 155 patients received balstilimab 3 mg/kg every 2 weeks alone or with zalifrelimab 1 mg/kg every 6 weeks for up to 2 years, respectively. ORR was 14% and 22%, with 2% and 6% of complete response for the single agent and the combination and DOR was 15.4 months and not reached, respectively. Treatment was well-tolerated in both studies, with 30% and 35% of patients experiencing all grades of immune-related AEs, and severe (grade 3+) AEs in 8% and 10.5% for mono and combo, respectively. No treatment-related deaths were observed with balstilimab, while two patients died for nephritis and pneumonitis in the combination treatment trial [54] (Table 1).

**Table 1:** Published clinical trials evaluating immune checkpoint inhibitors in the treatment of cervical cancer.

Ref.	Trial	Phase	Pts	Setting	Drugs and Schedule	Primary Endpoint	Results
[40,41]	Frenel et al., 2017 (Keynote 028)	Ib	24	Recurrent PD-L1 Positive	Pembrolizumab 10 mg/kg q2w for up to 2 years	ORR	17%
[42]	Chung et al., 2019 (Keynote 158)	II	98	Recurrent	Pembrolizumab 200 mg q3w for up to 2 years	ORR	12.2%
[43]	Naumann et al., 2019 (CheckMate 358)	I/II	19	Recurrent Metastatic HPV+	Nivolumab 240 mg q2w for up to 2 years	ORR	26.3%
[48]	Santin et al., 2019	II	26	Persistent Recurrent	Nivolumab 3 mg/kg q2w until PD or intolerable toxicity	ORR	4%
[49]	Lheureux et al., 2018	I/II	42	Metastatic	Phase I: Ipilimumab 3 mg/kg q3w for 4 cycles. Phase II: Ipilimumab 10 mg/kg q3w for 4 cycles followed by 4 cycles of maintenance therapy q12w	ORR and Safety	ORR 2.4% AEs ≥ Gr 3 were reported in 12 pts
[50]	Mayadev et al., 2017	I	34	FIGO stage IB2/IIA or IIB/IIIB/IVA and positive nodes	Weekly cisplatin (40 mg/m^2^) for 6 cycles and extended field radiation → sequential ipilimumab (3 dose levels: 3 mg/kg, 10 mg/kg and an expansion cohort of 10 mg/kg)	MTD and DLT	MTD = 10 mg/kg
[52]	Naumann et al., 2019 (CheckMate 358)	I/II	91	Recurrent Metastatic HPV+	Combo A: nivolumab 3 mg/kg q2w and ipilimumab 1 mg/kg q6w Combo B: nivolumab 1 mg/kg and ipilimumab 3 mg/kg q3w, for 4 doses followed by nivolumab 240 mg q2w for up to 2 years	ORR	Without PST: Combo A 32% Combo B 46% With PST: Combo A 23% Combo B 36%
[53]	Callahan et al., 2017	I	13 *	Non responders Relapsed	Durvalumab 1500 mg q4w and tremelimumab 75 mg q4w × 4	MTD and safety	Regimen used for expansion phase: Durvalumab 1500 mg q4w and tremelimumab 75 mg q4w × 4. TRAEs ≥ Gr 3 were reported in 12 pts
[54]	O’ Malley et al., 2020	II	161	Recurrent Metastatic	Balstilimab 3 mg/kg q2w up to 2 years	ORR	14%
			155	Recurrent Metastatic	Balstilimab 3 mg/kg q2w in combination with zalifrelimab 1 mg/kg q6w up to 2 years	ORR	22%

* evaluable; ORR = objective response rate, PD = progression disease, AEs = adverse events, Pts = patients, MTD = maximum tolerated dose, DLT = dose-limiting toxicities, PST = previous systemic therapy.

The data presented in all these published studies should be read in the context of second line chemotherapies efficacy, with response rates ranging between 5–29%, median PFS of 1.9–5.0 months, and median OS 5.0–12.7 months [11]. However, since these studies are early phase and non-randomized, it is difficult to reach conclusions, even though the results appear in many cases comparable or even better than the data with chemotherapy. Moreover, immunotherapy often shows an advantage in terms of survival, without an important reduction of tumor burden. Therefore, considering that the objective response, which is the primary endpoint of the majority of these trials, does not fully represents the potential of immunotherapy, this strategy appears promising, especially considering the good safety profile.

### 3.3. Future Developments of Immune Checkpoint Inhibitors in Cervical Cancer: Ongoing Clinical Trials

Considering the good results obtained in the early phase studies, several trials evaluating ICIs in the treatment of CC in various settings of disease are currently ongoing (Table 2).

#### 3.3.1. Locally Advanced Cervical Cancer

At least four trials are evaluating the possible role of ICIs in combination with CRT or neoadjuvant chemotherapy in locally advanced CC.

One of these studies, the phase III, randomized, multicenter, international, double-blind, placebo-controlled CALLA study (NCT03830866), is evaluating durvalumab in association and following concurrent CRT versus concurrent CRT alone. Patients receive radiotherapy with cisplatin or carboplatin once a week for 5 weeks, followed by brachytherapy, with durvalumab or placebo for 24 cycles. The primary endpoint is PFS. Patient enrollment is continuing globally until April 2024 [55].

Instead, pembrolizumab is being studied in the remaining three trials: in the first two studies always in association with CRT, while in the third trial in the neoadjuvant setting. In particular, in the first trial, the phase III, randomized, placebo-controlled ENGOT-cx11/KEYNOTE-A18 study (NCT04221945), pembrolizumab or placebo are given together with CRT, followed by 15 cycles of pembrolizumab or placebo every 6 weeks. Primary endpoints are PFS by blinded independent central review and OS [56].

The second trial is an open-label phase II study (NCT02635360), where pembrolizumab, administered sequentially or concurrently with standard CRT, is evaluated in 88 patients with locally advanced CC. The primary objectives are the safety and the evaluation of immune response to pembrolizumab, while secondary objectives are the study of metabolic response and the rates of distant metastases [57]. 

Finally, since recent evidences suggest that exclusive chemotherapy in the primary treatment of CC is a valuable option, this strategy merits further exploration. In particular, chemotherapy can be used in the neoadjuvant and adjuvant setting (when indicated according to post surgery risk factors), reserving radiotherapy for recurrences with the aim to avoid toxicity of radiation treatment, particularly in young patients. Unfortunately, response rate to neoadjuvant chemotherapy is about 80%, and 30% of patients will receive adjuvant radiotherapy or chemoradiation based on the risk factors emerging from the pathology report. For this reason, new drugs are required to potentiate the chemotherapy effect reducing the necessity of adjuvant radiotherapy and preserving patients’ quality of life. In this context, the single-arm phase II multicenter study MITO CERV-3 (NCT04238988) evaluates the neoadjuvant treatment with pembrolizumab, carboplatin, and paclitaxel in stage IB2-IIB CCs. After three cycles of treatment, in absence of progression disease, patients will undergo surgery; thereafter, those with high risk factors for relapse at pathologic evaluation, will receive three further cycles of the same treatment followed by maintenance with pembrolizumab for up to 35 cycles. The primary endpoint is 2 years PFS [58].

#### 3.3.2. Advanced Cervical Cancer: First Line Treatment

Three first-line phase III trials are evaluating the association between ICIs and chemotherapy.

The first study is Keynote-826 (NCT03635567), a phase III, randomized, double-blind, placebo-controlled trial evaluating pembrolizumab in association with chemotherapy (CT) for persistent, recurrent, or metastatic CC. In this trial, patients who have not received CT for recurrence and are not amenable to curative treatment are randomized to investigators’ choice CT with cisplatin or carboplatin and paclitaxel, with or without bevacizumab, plus pembrolizumab or placebo for up to 35 cycles. The primary endpoints are blinded independent central review PFS and OS [59].

The second is the BEATcc, ENGOT-Cx10/GEICO 68-C/GOG3030/JGOG1084 (NCT03556839) trial that is studying the role of immunotherapy with atezolizumab in association with cisplatin-paclitaxel and bevacizumab in the same setting. The primary endpoint is OS and mature data are expected in 2023 [60].

Furthermore, in recurrent, platinum resistant or metastatic CC, the open-label, randomized, phase III GOG 3016/ENGOT-cx9 trial (NCT03257267) evaluates the role of another PD-1 inhibitor, cemiplimab. In this study, patients receive monotherapy with cemiplimab or investigators’ choice chemotherapy for up to 96 weeks. The primary objective is OS and trial has currently completed recruitment [61].

#### 3.3.3. Advanced Cervical Cancer: Second-Line Treatment

In this setting, atezolizumab, alone or in combination with tiragolumab, a novel cancer immunotherapy designed to bind to T cell immunoreceptor with immunoglobulin and ITIM domain (TIGIT), recently being granted Breakthrough Therapy Designation (BTD) by the FDA in combination with atezolizumab for the first-line treatment of NSCLC, is under investigation in the phase II trial SKYSCRAPER-04 (NCT04300647). The purpose of this study is to evaluate the efficacy and safety of atezolizumab monotherapy or in combination with tiragolumab in patients with recurrent or metastatic PD-L1-positive CC. The primary endpoint is Independent Review Committee Assessed ORR [62].

### 3.4. New Immune Checkpoint Inhibitors Combinations

Researchers have explored whether the new combination strategies could ameliorate opportunity for patients treatment. 

In particular, a rationale exists for the combination of immunotherapy and antiangiogenic agents and preclinical models demonstrated that the inhibition of vascular endothelial growth factor (VEGF) pathway induces the immunity response against the tumor and improves the efficacy of immune checkpoint inhibition [63], and the association of ICIs and antiangiogenic agents led to a synergistic effect in vivo [64]. Two trials are currently evaluating ICIs and antiangiogenic agents combinations. The first is studying the combination of the anti-PD-1 camrelizumab with the tyrosine kinase inhibitor targeting VEGF receptor 2 apatinib in patients progressed after at least one line of systemic chemotherapy for metastatic, recurrent, or persistent CC. The primary endpoint is ORR and 55.6% of evaluable patients achieved an objective response, including two CR and 23 PR. Median duration of response and median OS were not reached and mPFS was 8.8 months. A total of 71.1% of patients had grade ≥3 treatment-related adverse events and the most frequent were hypertension (24.4%), anemia (20.0%), and fatigue (15.6%) [65]. The second is the FERMATA trial, a currently ongoing randomized double-blind study that evaluates BCD-100, a mAb directed against PD-1, or placebo in association with platinum-based chemotherapy with or without bevacizumab in first-line for patients with advanced CC [66]. 

Finally, since TGF-β appears to be a key mediator of resistance to anti-PD-(L)1 treatments [67], its inhibition in association to PD-(L)1 inhibitors seems a promising strategy. In particular, a first-in-class bifunctional fusion protein targeting both TGF- β and PD-L1 is currently under evaluation in a phase II trial [68], after the encouraging results of the phase I study that showed an ORR of 24% (8% CR and 16% PR) [69].

## 4. Conclusions

Despite the introduction in clinical practice of a specific vaccine and screening programs, CC remains one of the most common neoplasia in women worldwide, with a poor prognosis and a high mortality rate when diagnosed in an advanced or recurrent setting. 

Supporting the urgency of new and effective treatment strategies ICIs showed impressive results in the treatment of several cancers and demonstrated good tolerability. A strong rationale supports the use of ICIs in CC and preliminary trials showed encouraging results in terms of ORR, PFS, and OS, both with monotherapy [40,41,42,43,48,49,50] or association strategies [52,53,54], and, mostly, with a good safety profile. Several trials evaluating ICIs in CC treatment are now ongoing in different settings of disease and in different association regimens [55,56,57,58,59,60,61,62,65,66,68,69], and their results are anxiously awaited. These trials will define the role of this strategy in the treatment algorithm and will possibly identify predictive biomarkers of response, able to identify the populations who can benefit most (i.e., PD-L1-positive or HPV-positive tumors). 

## Figures and Tables

**Table 2:** Ongoing clinical trials evaluating immune checkpoint inhibitors in the treatment of cervical cancer.

Ref.	Trial	Phase	Pts	Setting	Drugs and Schedule	Primary Endpoint
[55]	NCT03830866 (CALLA)	III	714	Locally advanced	External beam radiotherapy with cisplatin (40 mg/m^2^) or carboplatin (AUC 2) once a week for 5 weeks, followed by brachytherapy, with durvalumab 1500 mg or placebo q4w for 24 cycles.	PFS
[56]	NCT04221945 (ENGOT-cx11/ KEYNOTE-A18)	III	980	Locally advanced	Pembrolizumab 200 mg or placebo q3w for 5 cycles + CRT (weekly cisplatin 40 mg/m^2^ + external beam radiotherapy followed by brachytherapy) followed by 15 cycles of pembrolizumab 400 mg or placebo q6w.	PFS and OS
[57]	NCT02635360	II	88	Locally advanced	Following chemoradiation Cisplatin 40 mg weekly and 4–6 fractions of brachytherapy radiation for 5–6 weeks → pembrolizumab 200 mg q3w for 3 months. Concurrent to chemoradiation Cisplatin 40 mg weekly and 4–6 fractions of brachytherapy radiation for 5–6 weeks and concurrent pembrolizumab 200 mg q3w for 3 months.	Immune Response and safety
[58]	NCT04238988 (CERV-3)	II	45	Locally advanced	3 cycles of NACT with carboplatin AUC 5, paclitaxel 175 mg/m^2^ and pembrolizumab 200 mg q3w → surgery → adjuvant carboplatin and paclitaxel in combination with pembrolizumab, followed by pembrolizumab 200 mg q3w for up to 35 cycles (only high risk)	2-years PFS
[59]	NCT03635567 (Keynote-826)	III	600	Persistent Recurrent Metastatic	Investigators’ choice CT (paclitaxel 175 mg/m^2^ + cisplatin 50 mg/m^2^ or carboplatin AUC 5, with or without bevacizumab 15 mg/kg) + pembrolizumab 200 mg or placebo q3w until disease progression, unacceptable toxicity or patient withdrawal for up to 35 cycles (~2 years)	PFS and OS
[60]	NCT03556839 (BEATcc)	III	404	Persistent Recurrent Metastatic	Arm A: Cisplatin 50 mg/m^2^ or carboplatin AUC 5 + paclitaxel 175 mg/m^2^+ bevacizumab 15 mg/kg q3W. Patients who achieve a CR after ≥6 cycles may be allowed to continue bevacizumab. Arm B: Cisplatin 50 mg/m^2^ or carboplatin AUC 5 + paclitaxel 175 mg/m^2^ + bevacizumab 15 mg/kg + atezolizumab 1200 mg q3W. Patients who achieve a CR after ≥6 cycles may be allowed to continue bevacizumab plus atezolizumab.	OS
[61]	NCT03257267 (GOG 3016/ENGOT-cx9)	III	590	Recurrent Metastatic	Experimental: Cemiplimab 350 mg q3w Control therapy: Investigators’ choice: - pemetrexed 500 mg/m^2^ q3w - topotecan 1 mg/m^2^ daily x5 days, q3w - irinotecan 100 mg/m^2^ days 1, 8, 15, and 22, followed by 2 weeks rest, for 42 days (6-week cycle) - gemcitabine 1000 mg/m^2^ days 1 and 8, q3w - vinorelbine 30 mg/m^2^ days 1 and 8, q3w. Treatments will be given IV for up to 96 weeks.	OS
[62]	NCT04300647 (SKYSCRAPER-04)	II	220	Recurrent Metastatic	Atezolizumab 1200 mg q3w alone or in combination with tiragolumab 600 mg q3w	ORR

AUC = area under the curve, PFS = progression free survival, OS = overall survival, NACT = neoaodjuvant chemotherapy, CT = chemotherapy, CR = complete response.
